# Vaccination with DC-SIGN-Targeting αGC Liposomes Leads to Tumor Control, Irrespective of Suboptimally Activated T-Cells

**DOI:** 10.3390/pharmaceutics16050581

**Published:** 2024-04-24

**Authors:** Aram M. de Haas, Dorian A. Stolk, Sjoerd T. T. Schetters, Laura Goossens-Kruijssen, Eelco Keuning, Martino Ambrosini, Louis Boon, Hakan Kalay, Gert Storm, Hans J. van der Vliet, Tanja D. de Gruijl, Yvette van Kooyk

**Affiliations:** 1 Department of Molecular Cell Biology and Immunology, Amsterdam UMC, Cancer Center Amsterdam, Amsterdam Institute for Infection and Immunity, Vrije Universiteit Amsterdam, 1081 HV Amsterdam, The Netherlands; 2 Department of Medical Oncology, Amsterdam UMC, Cancer Center Amsterdam, Amsterdam Institute for Infection and Immunity, Vrije Universiteit Amsterdam, 1081 HV Amsterdam, The Netherlands; 3 LIPOSOMA BV, Meerpaalweg 5, 1332 BB Almere, The Netherlands; 4 JJP Biologics, 00-728 Warsaw, Poland; 5 Department of Biomaterials Science and Technology, University of Twente, 7500 AE Enschede, The Netherlands; 6 Department of Surgery, Yong Loo Lin School of Medicine, National University of Singapore, Singapore 119228, Singapore; 7 LAVA Therapeutics, 3584 CM Utrecht, The Netherlands

**Keywords:** dendritic cell (DC), DC-SIGN targeting, vaccine, liposome, invariant natural killer cell (iNKT), alpha galactosylceramide

## Abstract

Cancer vaccines have emerged as a potent strategy to improve cancer immunity, with or without the combination of checkpoint blockade. In our investigation, liposomal formulations containing synthetic long peptides and α-Galactosylceramide, along with a DC-SIGN-targeting ligand, Lewis Y (Le^Y^), were studied for their anti-tumor potential. The formulated liposomes boosted with anti-CD40 adjuvant demonstrated robust invariant natural killer (iNKT), CD4^+^, and CD8^+^ T-cell activation in vivo. The incorporation of Le^Y^ facilitated the targeting of antigen-presenting cells expressing DC-SIGN in vitro and in vivo. Surprisingly, mice vaccinated with Le^Y^-modified liposomes exhibited comparable tumor reduction and survival rates to those treated with untargeted counterparts despite a decrease in antigen-specific CD8^+^ T-cell responses. These results suggest that impaired induction of antigen-specific CD8^+^ T-cells via DC-SIGN targeting does not compromise anti-tumor potential, hinting at alternative immune activation routes beyond CD8^+^ T-cell activation.

## 1. Introduction

The foundations of vaccination strategies against cancer trace back to the observations of Wilhelm Busch and Friedrich Fehleisen, who noted that tumors could spontaneously shrink after a superficial infection with *Streptococcus pyogenes* [1,2]. This later led William Coley to treat patients with heat-inactivated extracts of the same pathogen to boost immunity [3]. The concept of using vaccination as a method to combat cancer has been considered an effective approach for many years. However, its clinical effectiveness has been limited up to this point. On the other hand, immune checkpoint blockade (ICB) has been a story of success [4,5], but its efficacy has been limited to a relatively small subset of patients. Numerous approaches have been attempted to develop vaccines that can transform “cold” tumors (characterized by low immune cell activity) into ones with enhanced immune cell infiltration, thereby boosting the effectiveness of ICB therapy.

Since dendritic cells (DCs) play a pivotal role in the initiation and skewing of the immune response, it is beneficial for vaccines to target this immune subset directly. Currently, lipid nanoparticle vaccines containing modified mRNA have been effectively utilized in controlling the SARS-CoV-2 pandemic. However, the composition of these lipid nanoparticle vaccines leads to widespread biodistribution at both the organ and cellular levels, depending on the site of vaccination [6]. Therefore, targeting liposome vaccines to antigen-presenting cells (APCs) may allow for a reduction of dose and the reduction of off-target side effects, leading to increased effectivity. Lipid nanoparticle vaccines can be targeted to DCs by incorporating ligands that specifically bind to receptors on DCs, such as C-type lectin receptors (CLRs) [7,8]. The glycan Lewis Y (Le^Y^) specifically binds to the CLR Langerin and the dendritic cell-specific intercellular adhesion molecule-3-grabbing non-integrin (DC-SIGN), which are expressed by Langerhans cells (LC) and dermal DC (dDC) in human skin, respectively. Therefore, Le^Y^ can induce efficient binding and internalization of the vaccine [9]. As such, it is an attractive approach to target various human skin-residing APCs, such as LCs and dDCs, through the inclusion of Le^Y^ in vaccine platforms. Expanding on this, our group’s previous research has already shown that targeting Langerin and DC-SIGN on LC and dDC subsets in a human ex vivo skin model with Le^Y^-bearing vaccination platforms results in improved internalization and antigen-specific CD8^+^ T-cell responses [10]. Various DC-SIGN targeting vaccination platforms have also been tested in vivo in a mouse model that expresses the human DC-SIGN receptor under the murine CD11c promotor (hDC-SIGN) [11]. In this model, immunization with AMAX made of synthetic long peptides (SLPs) and Le^Y^ yielded increased antigen-specific CD8^+^ T-cells in blood and spleen, as well as the frequency of cytokine producing CD4^+^ and CD8^+^ T-cells [12]. In the same hDC-SIGN mouse model, vaccinating with a tumor-specific antigen coated with Lewis B (Le^B^), another glycan targeting DC-SIGN, combined with the depletion of regulatory T-cells (T_reg_) led to long-term regression of the tumor [13].

For effective anti-tumor immunity, both innate and adaptive players should be mobilized. Therefore, developing vaccines that activate invariant natural killer T-cells (iNKT) that bridge innate and adaptive responses is a promising strategy [14]. To achieve this, vaccine formulations containing α-Galactosylceramide (αGC), a strong iNKT-activating ligand, can be employed. iNKT activation, via the presentation of αGC by CD1d on DCs, in turn, also leads to, amongst other things, increased activation of DCs, NK cells, and cytotoxic CD8^+^ T-cells [14]. Because of these synergistic effects, we have already developed a liposomal formulation containing αGC, an SLP, and the DC-SIGN-targeting ligand Le^Y^ in in vitro and a human ex vivo skin model [15]. Liposomes containing the DC-SIGN-targeting ligand Le^Y^ combined with αGC and SLP were efficiently delivered to human monocyte-derived DCs (moDCs) and different dDC subsets in the human skin. Additionally, moDCs and dDCs loaded with this Le^Y^-modified liposomal nanovaccine resulted in increased activation of iNKT and antigen-specific CD8^+^ T-cells [15]. However, the synergistic effect of activating both CD8^+^ T-cells and iNKT-cells on anti-tumor immunity could not be explored in human in vitro and ex vivo models.

We here aimed to test the in vivo efficacy of our DC-SIGN-targeting liposomal nanovaccine containing Le^Y^, SLP, and αGC. In immunization studies, we found that although iNKT-cells are known for their capacity to cross-activate DCs, the addition of an anti-CD40 antibody (αCD40) as an adjuvant is essential for proper iNKT, CD4^+^, and CD8^+^ T-cell responses in vivo. Moreover, we observed an increased uptake of Le^Y^-modified liposomes in murine bone marrow-derived dendritic cells (BMDCs) and APCs within murine skin. However, in vivo, this did not translate into enhanced iNKT, CD4^+^ T-cell, or antigen-specific CD8^+^ T-cell responses post-immunization, although an αGC-dependent effect on iNKT proliferation and CD4^+^ activation could be observed. Surprisingly, Le^Y^-modified liposomes containing SLP and αGC did not succeed in boosting the induction of antigen-specific CD8^+^ T-cells. However, the impaired induction of antigen-specific CD8^+^ T-cells did not impact the outcome regarding tumor growth or survival of the mice in a therapeutic vaccination setting using a B16-OVA melanoma tumor model.

## 2. Material and Methods

### 2.1. Multi-Color Flow Cytometry

Flow cytometry analyses were conducted at the O2 Flow Facility, located at Amsterdam UMC, VUmc site, utilizing either an X20 Fortessa flow cytometer (BD Biosciences, Franklin Lakes NJ, USA) or an Aurora 4L (Cytek Biosciences, Amsterdam, The Netherlands). Details on the antibodies utilized can be found in the Supplementary Materials.

### 2.2. Mice

C57BL/6 wild-type or transgenic hDC-SIGN mice on a C57BL/6 background were raised at VU University’s animal facility in Amsterdam, Netherlands, under specific pathogen-free conditions. These mice were used at ages ranging from 8 to 16 weeks [11]. Both sexes of mice were included in the studies. All experimental procedures received approval from the Animal Welfare Body (IvD) at VU University and were conducted in compliance with both national and international standards and regulations under a project license reviewed by the Dutch Central Authority for Scientific Procedures on Animals (CCD).

### 2.3. Production of Vaccines

The synthesis of peptides, glycolipids, and liposomes, as previously detailed [15], is elaborated in the Supplementary Materials.

### 2.4. Binding ELISA

Liposomal formulations at a concentration of 100 µM were applied to 96-well Nunc Maxisorp plates (Thermofisher, Waltham, MA, USA) and left to coat in ethanol overnight. Subsequently, the plates were rinsed with TSM buffer (20 mM Tris-HCl (pH 7.4), 150 mM NaCl, 1 mM CaCl_2_, 1 mM MgCl_2_) and blocked using 2% fat-free BSA (Roche, Basel, Switzerland) for 30 min at 37 °C. The plates were then incubated with 2 µg/mL DC-SIGN-fc (produced in-house) or Le^Y^ antibody (Gene Tex, Irvine, CA, USA) at room temperature. Following a 2 h incubation, secondary antibodies, including goat anti-human-HRP IgG (Thermofisher) and goat anti-mouse IgM-HRP (Jackson, Baltimore Pike, PA, USA), were added and the plates were incubated for an additional 30 min at room temperature. After five washes with TSM buffer, a substrate buffer containing TMB (Sigma, St. Louis, MO, USA) was added. The reaction was terminated using a citric acid buffer, and optical density (OD) readings were taken at 450 nm.

### 2.5. BMDC Culture

Bone marrow cells harvested from hDC-SIGN transgenic mice were thawed using IMDM (Lonza, Basel, Switzerland) enriched with 10% FCS (Corning, New York, NY, USA), 50 U/mL penicillin, 50 µg/mL streptomycin (Thermofisher), 50 µM 2-mercaptoethanol (Gibco, Waltham, MA, USA), and Glutamax (Gibco). After washing, the cells were seeded in Petri dishes (Greiner Bio-One, Alphen aan den Rijn, The Netherlands) at a density of 3–5 × 10^6^ cells per dish in 10 mL of complete IMDM (Gibco) with 60 ng/mL X-63. The medium was refreshed on day 2 and day 5 with 60 ng/mL of X-63. Bone marrow-derived dendritic cells (BMDCs) from 7–8-day cultures were utilized in subsequent experiments. These BMDCs were characterized in flow cytometry as cells expressing CD11c^+^ and high levels of MHC-II.

### 2.6. Uptake in BMDCs

Liposomes were produced with the lipophilic dye DiD. BMDCs were collected and pre-treated with or without 10 µg/mL AZN-D1 at 37 °C for 30 min, followed by washing and incubation with 100 µM of the labeled liposomes. BMDCs were cooled on ice for 45 min before being collected for the initial time point (t = 0). The cells were subsequently moved to a 37 °C environment, and samples were taken at 15, 30, and 60 min, designated as t = 15, t = 30, and t = 60, respectively, and immediately cooled on ice. These cells were fixed using 1% PFA (Brunschwig Chemie, Amsterdam, The Netherlands); and stained with MHC-II, CD11c, AZN-D1, and fixable viability dye; and examined using a Fortessa LSR flow cytometer.

### 2.7. Uptake in APCs In Vivo

While under anesthesia with 2–3% isoflurane, mice received subcutaneous injections in the flank with 300 nmol of liposomal formulations dissolved in sterile PBS (Fresenius Kabi, Bad Homburg von der Höhe, Germany), supplemented with 25 µg of αCD40 monoclonal antibody (produced in-house). After 12 h, the animals were terminated, and tissues such as the spleen and skin-draining lymph node were harvested. To extract a skin sample from the injection site, an 8 mm punch biopsy tool (KAI Medical, Osaka, Japan) was used. Details on the tissue digestion and staining processes are provided in the Supplementary Materials.

In short, tissues were mechanically and enzymatically digested, where after, a multi-color immune cell panel, including AZN-D1 to stain DC-SIGN, was used to stain the single-cell suspensions. Cells were analyzed by flow cytometry using Aurora (Cytek). After measurements, samples were unmixed in Spectroflow (Cytek) and analyzed in OMIQ (Dotmatics, Boston, MA, USA). Following the gating strategy shown in Supplementary Figure S5, single-cell events could be subdivided into various immune cell subsets from which the intensity of the DC-SIGN expression and DiD signal was evaluated.

### 2.8. Immunization Studies

Mice received subcutaneous immunizations with liposomal formulations, dosed at either 300 or 500 nmol, in a sterile PBS solution, with or without added αCD40 monoclonal antibody (produced in-house). Blood samples were drawn from the mandibular vein 48 h post-immunization. After a week, the mice were terminated to collect spleen and blood samples. To prepare single-cell suspensions from the spleens, tissues were mechanically disrupted through a 70 µm filter that had been pre-wet, followed by lysis of red blood cells using ACK buffer (Gibco) and subsequent T-cell counting for additional experiments.

Blood was processed by centrifugation to separate plasma and lysing of red blood cells with ACK buffer. Cells from the spleen and blood were then stained for 30 min at 4 °C with antibodies targeting membrane proteins or for 45 min at room temperature with either OVA257-264-H2-Kb-PE tetramer and CD8α or with PE labeled PBS57 (an αGC analog) loaded CD1d tetramer. Hereafter, cells underwent an additional wash and were treated with directly labeled primary antibodies. Fixation of cells was carried out using 1–4% PFA for 15 min at 4 °C.

### 2.9. Ex Vivo Re-Stimulation of Splenocytes

Splenocytes harvested from immunized mice were plated at a concentration of 3 × 10^6^ cells per well in 96-well flat-bottom plates. They were then stimulated with either 100 µg (OVA 262–276) for CD4^+^ T-cell re-stimulation or 10 µg of peptide (OVA 257–264) for CD8^+^ T-cell re-stimulation. The cells underwent incubation periods of 5 h and 48 h for CD8^+^ and CD4^+^ T-cell re-stimulations, respectively. To facilitate intracellular cytokine accumulation, 1 µg/mL Brefeldin A (Sigma) was added during the final 5 h of each re-stimulation phase. Following this, cells were collected and labeled with antibody mixture. They were then fixed with 1–4% paraformaldehyde (PFA) and permeabilized using a solution of 0.5% saponin in 0.5% BSA/PBS buffer for 15 min. Intracellular cytokines were stained within this permeabilization buffer, and the cells were incubated at 4 °C for 30 min. The analysis was conducted using a Fortessa LSR instrument (BD Biosciences).

### 2.10. Tumor Challenge and Therapeutic Vaccination

Mice were anesthetized with 2–3% isoflurane and then subcutaneously inoculated with 2 × 10^5^ B16-OVA tumor cells. Seven days after this initial procedure, the mice were grouped randomly into different treatment cohorts. Each group received a therapeutic vaccine dose of 500 nmol liposomal formulations enhanced with 25 µg of αCD40. Starting two days post-vaccination, mice underwent a systemic treatment with 250 µg of anti-PD1 (αPD1) administered via intraperitoneal injection, continuing for two weeks. Fourteen days following the first vaccination, a booster shot was administered containing 1 µg SIINFEKL peptide with an additional 25 µg of αCD40.

Blood samples for tetramer analysis were collected seven days after the booster vaccination. Tumor dimensions were assessed thrice weekly using a digital caliper, and tumor volume was calculated using the formula: $\frac{4}{3}$ × π×abc (where a represents half the width of tumor, b represents half the length of tumor, and c represents average of a and b/2). The mice were humanely terminated when their tumor size exceeded 800 mm^3^.

### 2.11. Statistics

Statistical analyses were carried out using GraphPad Prism (version 8) software. To compare multiple groups, a one-way ANOVA was conducted, followed by a Tukey post hoc test. The data are presented as the mean ± standard deviation (SD) unless specified otherwise. Significance levels were marked as * for *p* < 0.05, ** for *p* < 0.002, *** for *p* < 0.0002, and **** for *p* < 0.0001.

## 3. Results

### 3.1. Supplementation of SLP-αGC Liposomes with αCD40 Is Necessary for iNKT Proliferation and Induction of Antigen-Specific T-Cells

**Figure 1:**
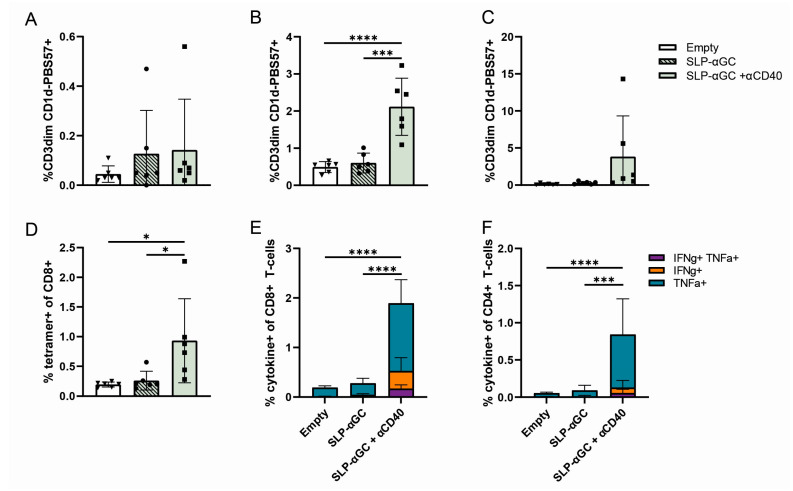
iNKT and T-cell activation by SLP- and αGC-containing liposomes is dependent on additional supplementation with αCD40. (**A**) Frequency of iNKT detected by CD1d-PBS57 staining in blood 48 h and (**B**) 7 days and (**C**) spleen 7 days after immunization with 300 nmol of liposomal formulations ± 25 µg αCD40. Cells were gated on live/single cells/CD45^+^/CD3dim/CD1d-PBS57^+^ (Figure S1). (**D**) Frequency of SIINFEKL^+^ (tetramer) CD8^+^ T-cells (Figure S2) in spleen 7 days post-immunization. (**E**) Intracellular cytokine detection in CD8^+^ and (**F**) CD4^+^ T-cells after peptide re-stimulation of splenocytes. Significance levels are marked as * for *p* < 0.05, *** for *p* < 0.0002, and **** for *p* < 0.0001.

We generated liposomes containing palmitoylated OTIOTII SLP, comprising both CD8 and CD4 epitopes of the model protein Ovalbumine (OVA), with the additional inclusion of αGC for iNKT activation. All liposomes used were similar in size (average 165 nm) and charge (Supplementary Table S1). We assessed whether SLP and αGC inclusion in liposomes synergize with αCD40, a well-known and often used adjuvant for activation of iNKT and T-cells in vivo [16]. To accomplish this, we compared s.c. immunization in hDC-SIGN transgenic mice using liposomal formulations, with and without the added supplementation of agonistic αCD40. Blood that was analyzed 48 h hours post-immunization showed no evident differences between frequencies of iNKT (Figure 1A and Figure S1). However, seven days post-immunization, we detected a significant increase in the frequency of iNKT in blood in mice immunized with SLP-αGC liposomes supplemented with αCD40 compared to other treatment groups, and a similar trend was observed in spleen (Figure 1B,C). Likewise, the frequency of antigen-specific (SIINFEKL) CD8^+^ T-cells seven days post-immunization was only increased significantly when SLP-αGC-containing liposomes were supplemented with αCD40 (Figure 1D and Figure S2). A similar effect was observed after ex vivo re-stimulation of splenocytes with the antigen; significant frequencies of (polyfunctional) cytokine-producing CD8^+^ and CD4^+^ T-cells were only detected in groups that received SLP-αGC liposomes plus αCD40 (Figure 1E,F). Altogether, we conclude that both αGC and SLP included in the liposomal formulations if supplemented with αCD40, were properly processed by the DC compartment in vivo to efficiently activate iNKT and T-cells. Therefore, all subsequent in vivo experiments were conducted in the presence of αCD40.

### 3.2. Addition of Le^Y^ to Liposomal Formulations Increases Uptake by DC-SIGN^+^ APCs In Vitro

**Figure 2:**
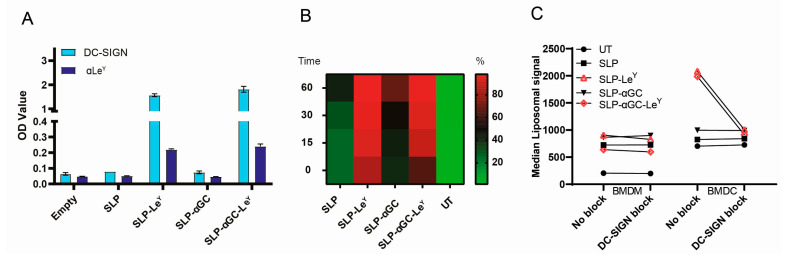
Le^Y^ incorporation in liposomes increases uptake by murine DCs via DC-SIGN. (**A**) Verification of the presence of Le^Y^ depicted as OD values obtained by binding ELISA using human recombinant DC-SIGN Fc and anti-Le^Y^ antibody (a Le^Y^). Data are representative of *n* = 2 performed in duplicate. (**B**) Frequency of DiD^+^ BMDC at different time points (t = 0 min, t = 15 min, t = 30 min, t = 60 min) post-incubation with 100 µM liposomes. Untreated (UT) BMDCs serve as control. Data are representative of *n* = 2 and shown as heatmap. (**C**) Effect of DC-SIGN block on uptake of liposomes by BMDCs (CD11c^+^, MHCII^++^) and BMDM (CD11c^+^, MHCIIint) after 60 min incubation at 37 degrees. Data are representative of *n* = 2.

Next, we aimed to further enhance the iNKT and T-cell responses by targeting our liposomal vaccines to the DC-SIGN receptor expressed on APCs. For this, we used the DC-SIGN glycan ligand Le^Y^, which was shown to enhance human iNKT and T-cell responses in vitro and ex vivo through binding this CLR [15]. As previously described, by palmitoylation of the glycan, we were able to insert Le^Y^ in the bilayer of our liposomal formulations (Supplementary Table S1) [15]. First, we confirmed the presence of Le^Y^ on liposomes through an ELISA via increased binding of DC-SIGN-Fc and anti-Le^Y^ antibodies (Figure 2A). To analyze whether the presence of Le^Y^ indeed leads to enhanced DC-SIGN targeting and uptake, we measured the uptake of fluorescently labeled (DiD) liposomes by DC-SIGN^+^ BMDC from hDC-SIGN transgenic mice (Figure S3) [17]. All formulations were taken up by BMDCs in a time-dependent manner, and Le^Y^ glycan-modified liposomes targeted BMDCs as measured by the increased frequency of DiD-positive BMDC (Figure 2B and Figure S4). Blocking DC-SIGN with the antibody AZN-D1 drastically decreased the overall uptake of Le^Y^ liposomes by these BMDCs, indicating that the uptake in BMDC, but interestingly not BMDM, was DC-SIGN-dependent (Figure 2C). In conclusion, modification of SLP-αGC liposomes with palmitic Le^Y^ leads to efficient targeting of DC-SIGN in BMDC and subsequent increased DC-SIGN mediated uptake.

### 3.3. Injection of Le^Y^-Coated Liposomes s.c. in Mice Specifically Target moDCs and cDC2s in the Skin

**Figure 3:**
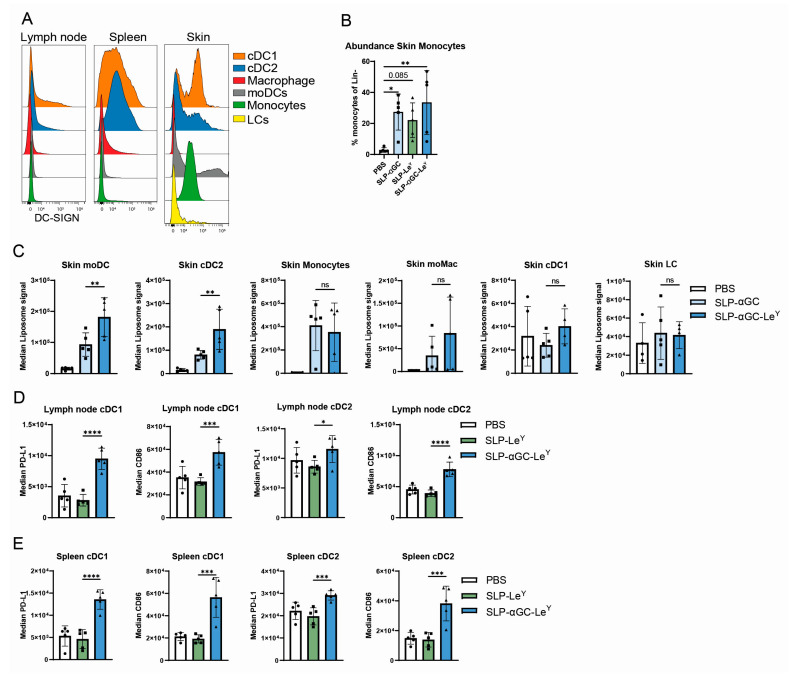
Le^Y^ αGC-modified liposomes target cDC2 and moDCs in the skin upon s.c. vaccination and induce maturation in lymph node and spleen. (**A**) expression of the human DC-SIGN receptor on APCs in lymph node, spleen, and skin. For gating strategy, see Figure S5. (**B**) Frequency of monocytes as percentage of the lineage negative (CD3, CD19, Ly6G) fraction 12 h after vaccination with 25 µg αCD40 and 300 nmol of liposomal formulations or PBS (*n* = 5). (**C**) Uptake of liposomes in APCs in the skin 12 h after vaccination. (**D**) Maturation of APCs in the skin draining lymph node or (**E**) spleen 12 h after vaccination measured by CD86 or PD-L1. Significance levels are marked as * for *p* < 0.05, ** for *p* < 0.002, *** for *p* < 0.0002, and **** for *p* < 0.0001.

After confirming that our Le^Y^-modified liposomes were functional in targeting APCs in vitro, we also wanted to assess their targeting efficacy in vivo. First, we validated the expression pattern of the human DC-SIGN receptor in our transgenic mouse model on various APCs in the skin, lymph nodes, and spleen by using a multi-color flow cytometry panel on single-cell suspensions of the various organs (Figure S5). Depending on the location, the DC-SIGN receptor is expressed on cDC1 (XCR1^+^), cDC2 (SIRP1α^+^), moDCs (CD11c^+^, Ly6C^+^) and macrophages (F4/80+) (Figure 3A and Figure S5). Next, we injected 300 nmol of liposomes or PBS s.c. in the flank of the mice, supplemented with the adjuvant αCD40. Twelve hours after vaccination, we observed an influx of monocytes at the site of vaccination; however, these cells did not take up the liposomes in a Le^Y^-dependent manner (Figure 3B,C). MoDCs, which differentiate from infiltrating monocytes, as well as cDC2s, showed increased uptake of the Le^Y^-coated liposomes over the control liposome in the skin, while this was not observed in cDC1, LCs (XCR1^−^, F4/80+, CD11b^+^), and MoMac (CD64^+^, CD11b^+^, MHCII^−^). Although we could not detect a significant liposomal signal in the skin-draining lymph node and spleen (Figure S6), we did observe an αGC-mediated maturation of APCs in these organs as measured by PD-L1 and CD86 (Figure 3D,E). Overall, we show efficient in vivo targeting of Le^Y^-αGC liposomes to DC-SIGN^+^ cDC2 and moDCs in the skin together with systemic maturation of cDC1 and cDC2 in the lymph node and spleen.

### 3.4. Incorporation of Le^Y^ into Liposomal Formulations for Increased DC-SIGN Targeting Fails to Further Increase iNKT, CD4^+^ T-Cells or Antigen-Specific CD8^+^ T-Cell Responses In Vivo

**Figure 4:**
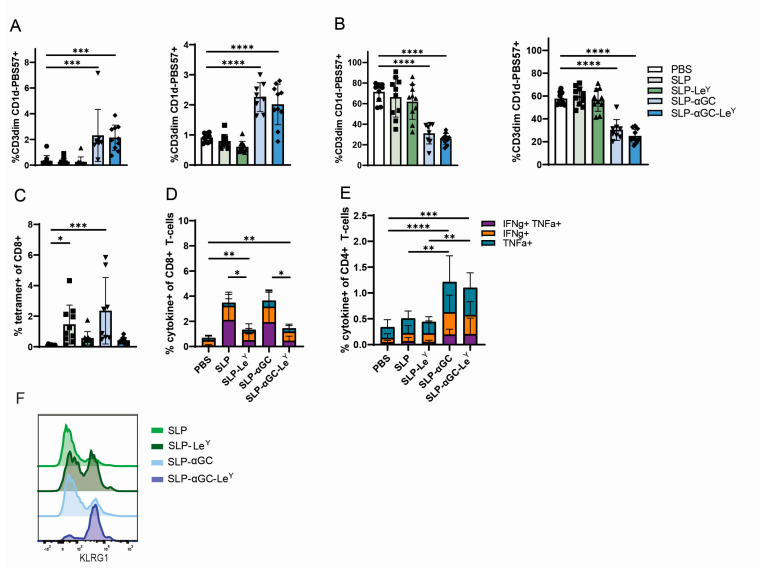
Immunization of mice with DC-SIGN-targeting Le^Y^-modified liposomal formulations has no beneficial effect on iNKT expansion or CD4^+^ T-cell activation and fails to induce antigen-specific CD8^+^ T-cell activation. (**A**) Frequency of iNKTs detected in blood (**left** panel) and spleen (**right** panel) 7 days post-s.c. immunization with 500 nmol liposomes containing SLP, Le^Y^, and αGC plus αCD40. (**B**) Frequency of NK1.1^+^ iNKTs in blood (**left** panel) and spleen (**right** panel) 7 days post-immunization. (**C**) Frequency of SIINFEKL^+^ CD8^+^ T-cells in spleen 7 days after liposomal immunization. (**D**) Detection of intracellular cytokines in CD8^+^ and (**E**) CD4^+^ T-cells after re-stimulation of splenocytes with cognate antigen peptide. (**F**) KLRG1 expression on SIINFEKL^+^ CD8^+^ T-cells in spleen 7 days after immunization. Data are shown as pooled data from two independent experiments with *n* = 5 mice per experiment. Significance levels are marked as * for *p* < 0.05, ** for *p* < 0.002, *** for *p* < 0.0002, and **** for *p* < 0.0001.

Next, we determined if increased targeting of our SLP-αGC-Le^Y^ liposomes in vivo would lead to increased iNKT and CD4^+^ and CD8^+^ T-cell responses in vivo. Hereto, hDC-SIGN mice were immunized s.c. with liposomal formulations containing various combinations of SLP, αGC, and the DC-SIGN-targeting moiety Le^Y^. As hypothesized based on the activation status of DCs upon vaccination (Figure 3D,E), only liposomes containing αGC induced substantial proliferation of iNKT (Figure 4A). Contradictory to our expectations, the inclusion of Le^Y^ to SLP-αGC liposomes did not further enhance iNKT expansion seven days after injection, as indicated by the equal frequencies of iNKT detected in blood and spleen. Additionally, the downregulation of NK1.1, which serves as an indicator of iNKT activation, was observed only after immunization with αGC containing nanoparticles and was also not influenced by the addition of Le^Y^ (Figure 4B). More striking, the targeted liposomes failed to induce antigen-specific T-cell responses since only SLP and SLP-αGC liposomes showed significant percentages of SIINFEKL^+^ CD8^+^ T-cells in the blood and spleen (Figure 4C). This finding was consistent with ex vivo re-stimulation of splenocytes, which revealed that Le^Y^ liposomes failed to induce a significant amount of effector cytokine (IFNγ and TNFα) producing CD8^+^ T-cells (Figure 4D). Furthermore, the addition of αGC and the subsequent iNKT proliferation did not appear to influence frequencies of antigen-specific CD8^+^ T-cells, as the number of SIINFKEKL^+^ CD8^+^ T-cells was equivalent in mice immunized with either SLP or SLP-αGC liposomes (Figure 4C). Interestingly, antigen-specific CD8^+^ T-cells in the Le^Y^ vaccinated groups had higher expression of the short-lived effector cells (SLEC) marker KLRG1 (Figure 4F). On the other hand, CD4^+^ T-cell activation seemed to depend on αGC mediated iNKT proliferation and was not negatively impacted by the inclusion of Le^Y^ into the liposomal formulations (Figure 4E). Overall, we concluded that modification of liposomes with Le^Y^ in vivo did not improve the iNKT or CD4^+^ T-cell responses and even hampered the priming of antigen-specific CD8^+^ T-cells but increased their KLRG1 expression.

### 3.5. Liposome Formulations Provide Similar Tumor Control, despite Lower Frequencies of Antigen-Specific CD8^+^ T-cells in Le^Y^ Groups

**Figure 5:**
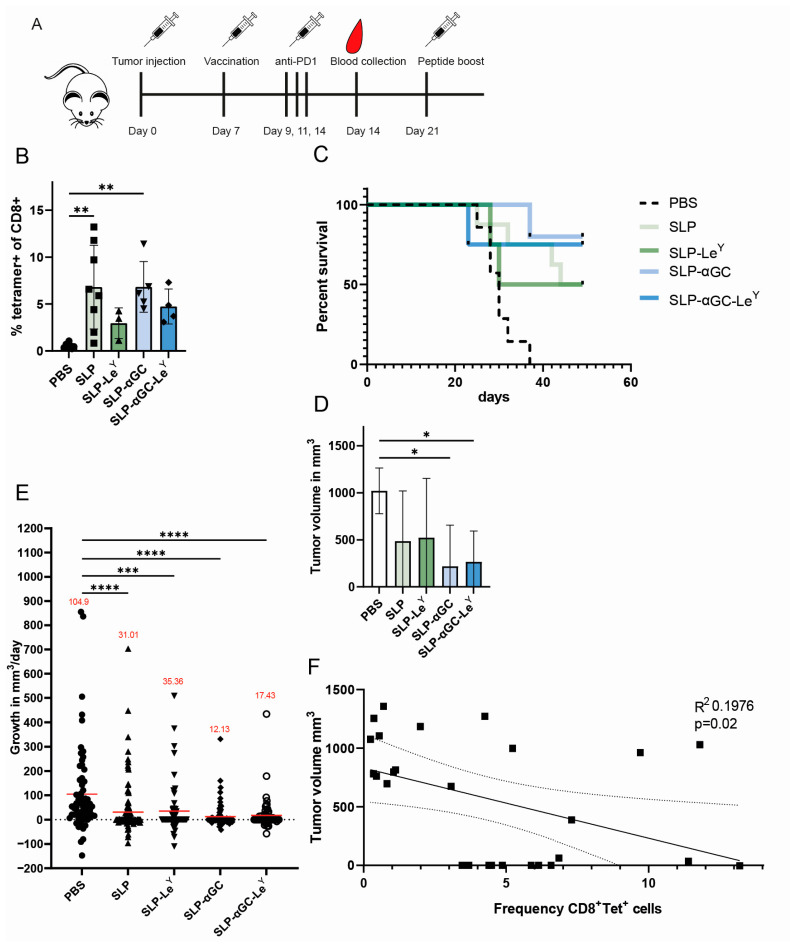
Therapeutic vaccination of mice bearing B16-OVA tumors is effective in all treatment groups, even though Le^Y^-modified liposomes fail to induce antigen-specific CD8^+^ T-cells. (**A**) Schematic overview of experimental setup. Mice were inoculated s.c. with 2 × 10^5^ B16-OVA tumor cells and seven days later vaccinated with 500 nmol of the liposomal formulations + 25 µg αCD40. (**B**) Frequency of SIINFEKL^+^ CD8^+^ T-cells seven days post-therapeutic vaccination. (**C**) Survival of mice over time after tumor inoculation and vaccination with liposomes. (**D**) Mean tumor volume per group of animals that developed a tumor at the final measurement. (**E**) Growth of tumor in mm^3^/day represented as ∆ growth of subsequent tumor measurements from all tumors in the indicated experimental group. Mean growth in mm^3^/day per measurement is indicated in red. (**F**) Correlation of frequency of SIINFEKL^+^ CD8^+^ T-cells versus tumor volume at last measurement. Significance levels are marked as * for *p* < 0.05, ** for *p* < 0.002, *** for *p* < 0.0002, and **** for *p* < 0.0001.

Finally, we evaluated the anti-tumor efficacy of targeted versus untargeted liposomal formulations in a therapeutic vaccination setting combined with αPD1 ICB. Mice were s.c. inoculated with 2 × 10^5^ B16-OVA tumor cells. Seven days after tumor injection, when palpable tumors were present, treatment with the various liposomal formulations was initiated (Figure 5A). Of note, due to the low tumor take, only four mice could be included in some treatment groups. Also here, seven days post-vaccination, animals vaccinated with Le^Y^ liposomes failed to induce significant induction of circulating antigen-specific CD8^+^ T-cells (Figure 5B). Despite the fact that CD8^+^ T-cells were lower in frequency, vaccination with Le^Y^-modified liposomal formulations increased the survival of mice (Figure 5C) and was effective in reducing the growth of B16-OVA tumors compared to the PBS control group (Figure 5D,E). This indicates that anti-tumor responses only weakly correlated with frequencies in the blood of antigen-specific CD8^+^ T-cells (Figure 5F). Furthermore, αGC-containing liposomes, irrespective of the targeting ligand, showed a tendency towards increased anti-tumor effect compared to liposomes without αGC (Figure 5C–E), potentially indicating an additional effect of αGC inclusion. In conclusion, although Le^Y^-modified liposomes induced lower induction of systemic antigen-specific CD8^+^ T-cells, these nanoparticles showed equal efficacious tumor control compared to their untargeted counterparts.

## 4. Discussion

In this study, we investigated whether we could improve the anti-tumor potential of therapeutic vaccination with αGC- and SLP-containing liposomes by targeting them to APCs via the DC-SIGN-binding moiety Le^Y^. In a B16-OVA mouse model, we demonstrated that our liposomal vaccine platform composed of the iNKT activator αGC, DC-SIGN-targeting glycan Le^Y^, and T-cell epitope containing SLP, in combination with αPD1 and αCD40, is able to decrease tumor outgrowth. Thereby, the component αGC induced systemic maturation of cDCs and mediated enhanced CD4^+^ T-cell and iNKT-cell engagement. Furthermore, we showed that the APC-targeting glycan Le^Y^ increases the uptake of our vaccine by APCs in vitro and in vivo; although this did not translate to increased T-cell responses in vivo, anti-tumor potential was retained. 

Surprisingly, we observed a limited induction of antigen-specific CD8^+^ T-cells after vaccination with Le^Y^ liposomes, even though the anti-tumor effect of these liposomes with systemic αPD1 was not impaired (Figure 5B,C). This questions whether the frequency of antigen-specific CD8^+^ T-cells in the blood upon vaccination is an adequate predictor for in vivo anti-tumor immunity [18]. Similar to our previous work, increased levels of antigen-specific CD8^+^ T-cells induced by a DC-SIGN-targeting OVA-Le^B^ vaccine did not enhance the anti-tumor effect; only in combination with T_reg_ depletion was an anti-tumor effect observed [13]. Similarly, Grabowska et al. found that enhancing CD8^+^ T-cell priming by targeting antigen-loaded nanoparticles to CD169^+^ macrophages also failed to enhance the anti-tumor effect [19]. As the underlying mechanisms for the above-mentioned discrepancies are unclear, a more elaborate immunological phenotyping is warranted to unravel the (cor)relation between immune activation status and anti-tumor effect, in which other immune cells besides CD8^+^ T-cells undoubtedly play important roles [20].

One potential mechanism by which tumor control is still exerted whilst lower SIINFEKL tetramer^+^ T-cells are present can be via epitope spreading, during which other immune-dominant TAAs induce antigen-specific immune responses [21]. Additionally, measuring tumor-specific T-cells in blood might be unrepresentative for T-cells that have already homed to the tumor itself. Moreover, the kinetics of T-cell proliferation might play a role, as we might have missed the optimum window for CD8^+^ T-cell proliferation in the Le^Y^ groups by measuring only at day seven instead of over multiple time points. An alternative explanation could be that different, more effective T-cell phenotypes are induced in the Le^Y^ groups, as seen by higher KLRG-1 expression (Figure 4F). KLRG1 expression is associated with effector T-cell differentiation, and these cell types are able to give rise to effector memory phenotypes as well [22,23,24]. Therefore, further research should include measurements for other TAAs (Trp2 or gp100) as well as more sample locations, time points, and phenotypic markers (such as PD-1 or CD127) for the effectiveness of T-cells.

To our surprise, we found a decreased induction of antigen-specific CD8^+^ T-cells after immunization with liposomal constructs containing Le^Y^ (Figure 4C). These results are in contrast to previous studies, in which immunization with either Le^B^- or Le^Y^-modified peptides or proteins increased antigen-specific CD8^+^ T-cells compared to unmodified products [12,13]. Of note, other DC-SIGN targeting studies used DC-SIGN-binding glycans that were directly conjugated to the antigens. In the current study, Le^Y^ had no direct association since it was separately palmitoylated and incorporated into the liposomal bilayer of the liposome. Moreover, the palmitoylation of the antigen itself has been shown to affect its processing as well [25]. Thereby, the incorporation of the three additional molecules in the liposome might lead to a suboptimal binding capacity of the Le^Y^ glycan because of steric hindrance. Overall, it appears that the chemical configuration of the antigen, targeting ligand, and platform may determine the final kinetics and/or outcome of the antigenic immune response.

Using the skin as an injection site for vaccines presents an appealing approach to stimulate immune responses, given the presence of numerous APCs within this organ. In our research, we observed that the uptake of liposomes was high in magnitude in all APCs in the skin, but only in moDCs and cDC2, unlike macrophages, monocytes, and cDC1, liposomes showed a Le^Y^ targeting effect (Figure 4C). We observed a small but insignificant amount of liposomal signal in the skin-draining lymph node (Figure S6). Changing the size of the nanoparticles has been shown to improve their drainage, which could lead to more optimal immune responses [26]. Alternatively, APCs that do take up the nanoparticles in the skin need longer than 12 h to migrate to the skin-draining lymph node or cannot migrate at all. Remarkably, irrespective of the low liposomal signal in the lymph node and spleen, we observed the maturation of the cDCs in these organs, indicating that the αGC still exerts a potent effect here (Figure 3D,E).

To conclude, we showed that by using a multi-component vaccine (including a DC-targeting, iNKT activator, and SLP moiety), we can confer effective anti-tumor immunity. Furthermore, our observations indicated that lower frequencies of antigen-specific CD8^+^ T-cells following vaccination with DC-SIGN-targeting Le^Y^-modified liposomes did not adversely affect the suppression of tumor growth. This suggests that another effector mechanism is involved, where the presence or absence of CD8^+^ T-cells does not solely determine the anti-tumor effect. Due to the complexity of the immune system and its responses to vaccines, future research should entail a multifactorial approach to better predict the effectiveness of our DC-SIGN-targeting cancer vaccines.

## Data Availability

The data presented in this study are available in this article and Supplementary Materials.

## Supplementary Materials

The following supporting information can be downloaded at https://www.mdpi.com/article/10.3390/pharmaceutics16050581/s1, File S1: Supplementary materials & methods; Figure S1: Gating strategy for iNKT-cells; Figure S2: Gating strategy for SIINFEKL^+^ CD8^+^ T-cells; Figure S3: Expression of DC-SIGN on BMDC and BMDM of hDC-SIGN mice; Figure S4: Representative plots and histograms of DiD signal in BMDCs after 60 min of incubation; Figure S5: (A) Gating strategy for APCs in lymph node. Cells were gated on: stable flow, no debris, single cells, Alive and CD45^+^, lineage negative (CD3^−^, CD19^−^, Ly6G^−^). Afterwards, APC subsets were identified as moDCs (CD11c^+^ Ly6C^+^), Macrophages (CD64^+^, F4/80^+^), Monocytes (CD11b^+^, Ly6C^+^/^++^), cDC1 (CD11c^+^ MHCII^+^, XCR1^+^) and cDC2 (CD11c^+^ MHCII^+^, Sirp1a^+^). Gating of subsets was validated with tSNE. (B) Gating strategy for APCs in spleen. Cells were gated on: stable flow, no debris, single cells, Alive and CD45^+^, lineage negative (CD3^−^, CD19^-^, Ly6G^−^). Afterwards, APC subsets were identified as moDCs (CD11c^+^ Ly6C^+^), Macrophages (F4/80^+^), Monocytes (CD11b^+^, Ly6C^+^/^++^), cDC1 (CD11c^+^ MHCII^+^, XCR1^+^) and cDC2 (CD11c^+^ MHCII^+^, Sirp1a^+^). Gating of subsets was validated with tSNE. (C) Gating strategy for APCs in skin. Cells were gated on: stable flow, no debris, single cells, unsaturated DiD signal, Alive and CD45^+^, lineage negative (CD3^−^, CD19^−^, Ly6G^−^). Afterwards, APC subsets were identified as Monocytes (CD64^−^, Ly6C^+^), monocyte derived macrophages (CD11b^+^ CD64^+^, MHCII^−^), monocyte derived DCs (CD11b^+^ CD64^+^, MHCII^+^), cDC1 (CD11c^+^ MHCII^+^, XCR1^+^), LCs (CD11c^+^ MHCII^+^, XCR1^−^, F4/80^+^) and cDC2 (CD11c^+^ MHCII^+^, XCR1^−^, CD11b^+^); Figure S6: MFI of liposomal signal in APCs in the skin draining lymph node 12 h after vaccination; Table S1: Mean size, polydispersity index, and Z potential with SD of five different batches of liposomes. References [15,27,28,29] are cited in the Supplementary Materials.
