# Supplementary material for: Vaccination with DC-SIGN-Targeting αGC Liposomes Leads to Tumor Control, Irrespective of Suboptimally Activated T-Cells
Source: Pharmaceutics. 2024 Apr 24;16(5):581. doi: 10.3390/pharmaceutics16050581 (PMC11124829; doi:10.3390/pharmaceutics16050581)
Supplement: Supplementary file 1 [file pharmaceutics-16-00581-s001.zip › Supplementary figures.pdf]

supplementary figures

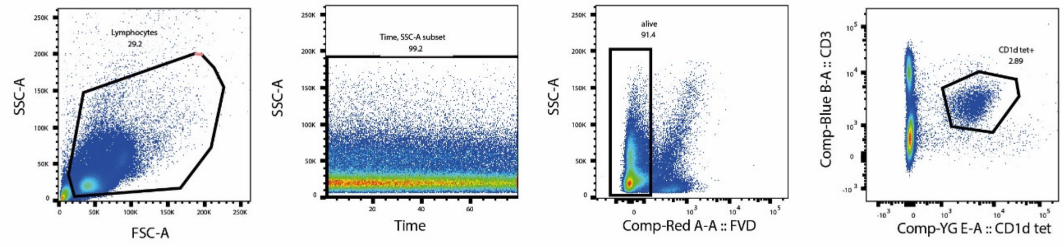

Figure S1. Gating strategy for iNKT cells.

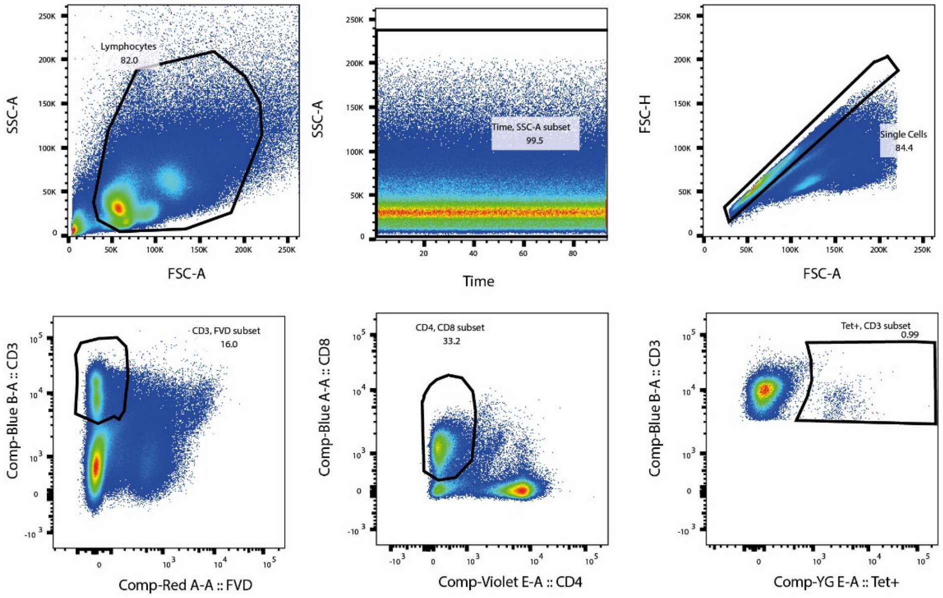

Figure S2. Gating strategy for SIINFEKL+ CD8 T-cells.

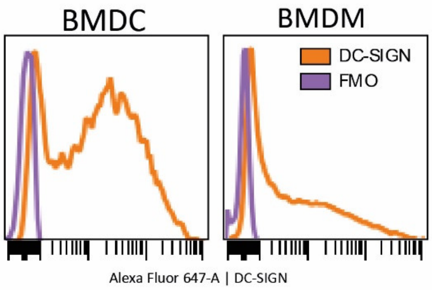

Figure S3. Expression of DC-SIGN on BMDC and BMDM of hDC-SIGN mice.

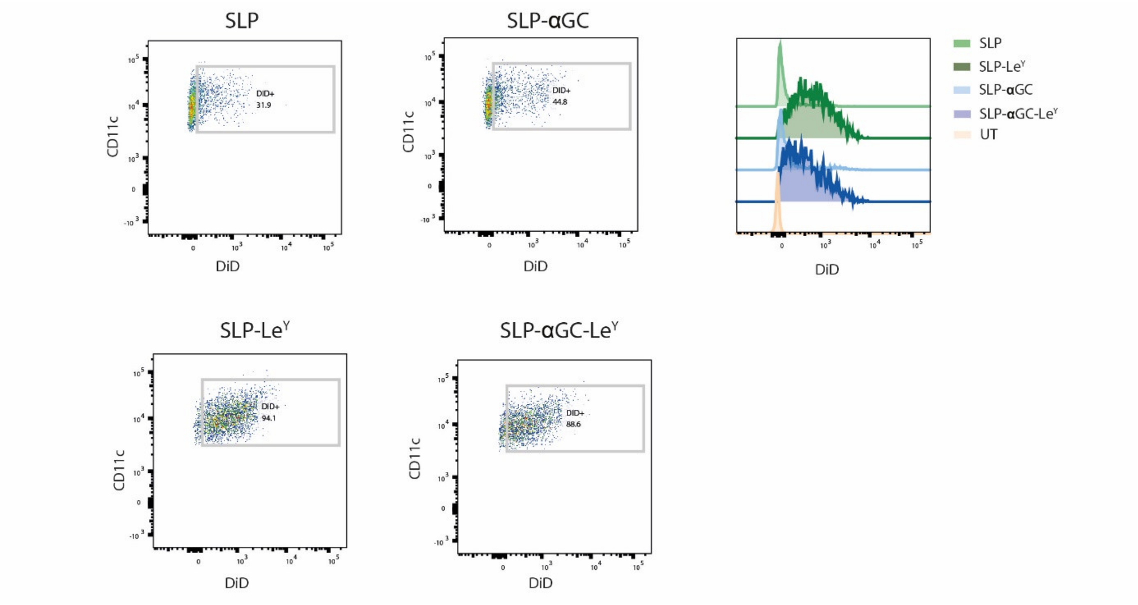

Figure S4. Representative plots and histograms of DiD signal in BMDCs after 60 minutes of incubation.

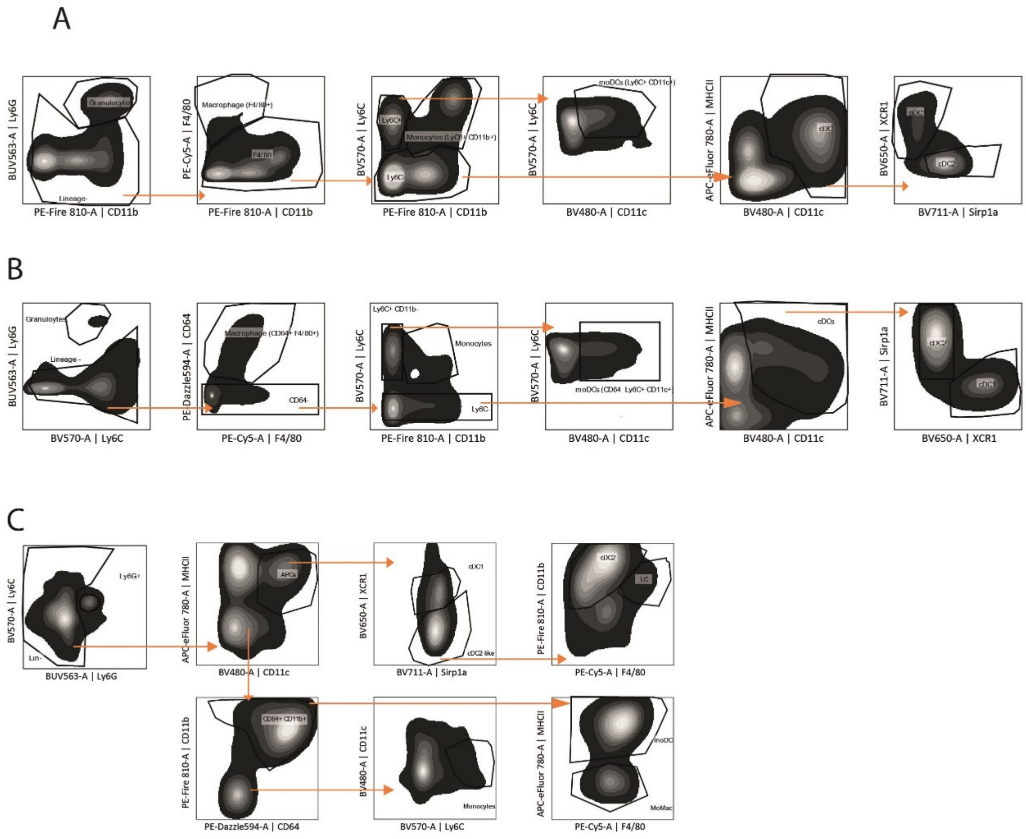

Figure S5. (A) Gating strategy for APCs in lymph node. Cells were gated on: stable flow, no debris, single cells, Alive and CD45^{+}, lineage negative (CD3^{-}, CD19^{-}, Ly6G^{-}). Afterwards, APC subsets were

identified as moDCs ($CD11c^+$ Ly6C+), Macrophages (CD64+, F4/80+), Monocytes (CD11b+, Ly6C+/++), cDC1 (CD11c+ MHCII+, XCR1+) and cDC2 (CD11c+ MHCII+, Sirp1a+). Gating of subsets was validated with tSNE (data not shown). (B) Gating strategy for APCs in spleen. Cells were gated on: stable flow, no debris, single cells, Alive and $CD45^+$, lineage negative ($CD3^-$, $CD19^-$, $Ly6G^-$). Afterwards, APC subsets were identified as moDCs ($CD11c^+$ Ly6C+), Macrophages (F4/80+), Monocytes (CD11b+, Ly6C+/++), cDC1 (CD11c+ MHCII+, XCR1+) and cDC2 (CD11c+ MHCII+, Sirp1a+). Gating of subsets was validated with tSNE (data not shown). (C) Gating strategy for APCs in skin. Cells were gated on: stable flow, no debris, single cells, unsaturated DiD signal, Alive and $CD45^+$, lineage negative ($CD3^-$, $CD19^-$, $Ly6G^-$). Afterwards, APC subsets were identified as Monocytes (CD64-, Ly6C+), monocyte derived macrophages (CD11b+ CD64+, MHCII-), monocyte derived DCs (CD11b+ CD64+, MHCII+), cDC1 (CD11c+ MHCII+, XCR1+), LCs (CD11c+ MHCII+, XCR1-, F4/80+) and cDC2 (CD11c+ MHCII+, XCR1-, CD11b+). Gating of subsets was validated with tSNE (data not shown).

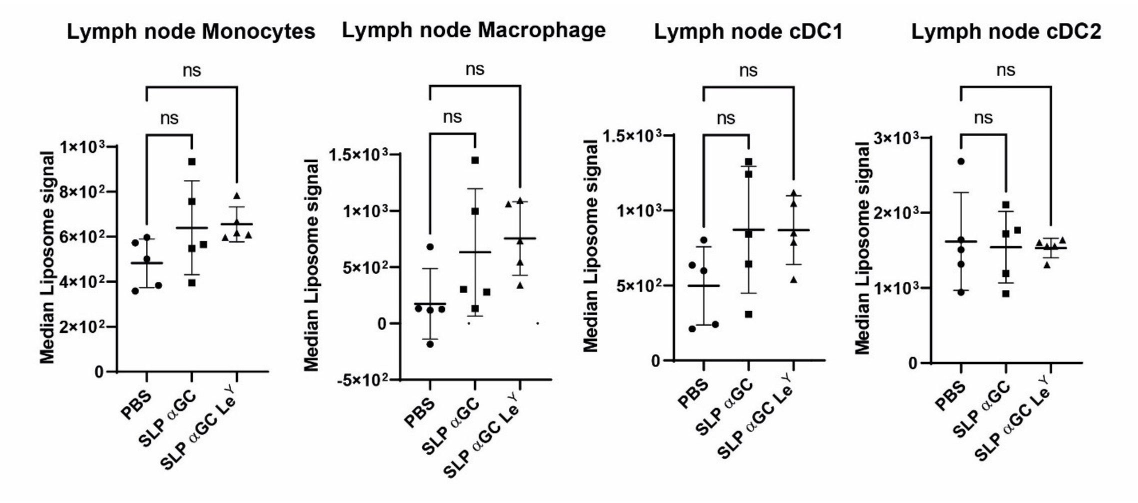

Figure S6. MFI of liposomal signal in APCs in the skin draining lymph node 12 hours after vaccination.
