# Supplementary material for: Vaccination with DC-SIGN-Targeting αGC Liposomes Leads to Tumor Control, Irrespective of Suboptimally Activated T-Cells
Source: Pharmaceutics. 2024 Apr 24;16(5):581. doi: 10.3390/pharmaceutics16050581 (PMC11124829; doi:10.3390/pharmaceutics16050581)
Supplement: Supplementary file 1 [file pharmaceutics-16-00581-s001.zip › Supplementary materials & methods.pdf]

Supplementary Material and Methods

Peptide synthesis and characterization

The antigenic peptide OTIOTII (EEKSIINFEKLISQAVHAAHAEINEAGRKEEK) was synthesized as described before [14]. In short, we made use of solid phase peptide synthesis using Fmoc chemistry on a Symphony peptide synthesizer (Protein Technologies Inc., Tucson, U.S.). A palmitic tail was added at the N-terminus of the OTIOTII peptide through a reaction with palmitic anhydride in dichloromethane, resulting in palmitoylated SLP (from here on, shortly, SLP). Lipopeptides were cleaved and purified on a preparative Ultimate 3000 HPLC system (Thermofisher) over a VYDAC 214MS1022 C4 25 × 250 mm column (Grace Davidson). Mass and purity were confirmed by UPLC-MS on a Ultimate 3000 UHPLC system (Thermofisher) hyphenated with a LCQ-Deca XP Iontrap ESI mass spectrometer (Thermofinnigan) using a Prosphere HP C4 5 µm 150 × 4.6 mm column and ionizing the sample in positive mode.

Lewis^{Y} glycolipid synthesis

Le^{Y}-hexadecanohydrazide (palmitic hydrazide) ea. palmitoylated Le^{Y} (from here on, shortly, Le^{Y}) was prepared as described before [14]. In short, it was prepared from Le^{Y} pentasaccharide (Elicityl, Crolles, France) and hexadecanohydrazide through a reductive amination reaction. $CHCl_3$/MeOH/H_2O was added at 8:1:8 v/v ratio. The white slurry extracted from the interphase was freeze-dried to remove residual solvent. ESI-MS (ThermoFinnigan LCQ-Deca XP Iontrap mass spectrometer in positive mode) using nanospray capillary needle was used to confirm the correct mass of glycolipid.

Generation of liposomes

Liposomes containing palmitoyl-OTIOTII SLP, αGC and Le^{Y} targeting glycan were generated as described previously [14]. In short, liposomes were prepared from a mixture of phospholipids and

cholesterol utilizing the film extrusion method. Briefly, EPC-35 (Lipoid):EPG-Na (Lipoid):Cholesterol (Sigma-Aldrich, St. Louis, U.S.) at a ratio of 3.8:1:2.5 in mol were dissolved in a mixture of chloroform/methanol. The lipophilic fluorescent tracer DiD (1′-dioctadecyl-3,3,3′,3′-tetramethyl indodicarbocyanine; Life Technologies, Frederick, U.S. 0.1% in mol) was added to the mixture (where indicated) as well as palmitoyl-OTIOTII (800µg), Le^{Y} (1.5mg) and the iNKT cell activator αGC, KRN7000 (Funakoshi, Tokyo, Japan) (12µg) and extruded trough two stacked polycarbonate filters of 200nm. Size, polydispersity index and zeta potential was determined prior to use, as previously described [26]. After destruction with perchloric acid, phospholipid content (concentration in molar) was quantified spectrophotometrically as described before [27]. The liposome dosage used in the experiments was calculated based on the phospholipid contents in mol as previously described [28].

Preparation of single cell suspensions and staining

Spleen, lymph node and skin were first mechanically dissociated. Thereafter, spleens were digested with 3mg/ml lidocaine (Sigma Aldrich), 2 Wünsch units/mL Liberase TL (Roche, Mannheim, Germany) and 50 mg/mL DNase I (Roche) at 37 ˚C for 12 minutes. The digestion was stopped with the addition of ice-cold RPMI-1640 medium (Gibco, Life Technologies) containing 10% FCS (Biowest), 50 µM 2-mercaptoethanol, 20 mM HEPES and 10 mM ethylenediaminetetraacetic acid (EDTA). Red blood cells were lysed with ACK buffer (Gibco), and cells were filtered through a 70µm filter afterwards. Lymph nodes were further digested by incubating them in 100µg/ml Liberase TL (Roche) with 50µg/ml DNAse I (Roche) in PBS at 37 ˚C for 30 minutes. The mechanically disrupted skin was further digested with 2 Wünsch units/mL Liberase TL (Roche) and 50 mg/mL DNase I (Roche) at 37 ˚C for 25 minutes. Both the digestion reaction of the lymph node and skin were stopped with the ice-cold RPMI-1640 medium as above. Similarly, those organs were filtered over a 70µm filter. Cells were stained in the first step with Life Dead Blue and Fc-block. Thereafter the cell suspensions were stained with Ly6G, CD19, CD3, PD-L1, CD11c, Ly6C, XCR1, Sirp1a, CD86, AZN-D1, CD64, F4/80, CD45, MHCII, CD11b and

after that fixated with 2% PFA (Brunschwig). Samples were measured on Aurora 4L (Cytek Biosciences).

Antibodies

Fixable Viability dye eFluor 780 (eBioscience, San Diego, U.S.), $OVA_{257\text{-}264}$-H2-Kb-PE tetramers a kind gift from Dr. J.W. Drijfhout (LUMC, Leiden, Netherlands), anti-CD3 (KT-3, in-house), anti-CD4 (RM4-5, eBioscience, & GK1.5, Invitrogen, Waltham, U.S.), anti-CD8a (53-6.7, Biolegend, San Diego, U.S.), anti-NK1.1 (PK136, Biolegend), PBS-57 loaded CD1d was kindly provided by NIH, anti-TNFa (TN3-19, eBioscience), anti-IFNg (XMG1.2, eBioscience), anti-CD44 (IM7, eBioscience), anti-CD62L (MEL-14, eBioscience), anti-KLRG1 (2F1/KLRG1, Biolegend), anti-MHCII (M5/114.15.2, BD Biosciences), anti-CD11c (N418, Biolegend), anti-Ly6G (1A8, BD Biosciences), anti-CD19 (1D3, BD Biosciences), anti-CD3 (17A2 BD Biosciences), anti-PDL1 (MIH5, Invitrogen), anti-CD11c (HL3, BD Biosciences), anti-Ly6C (HK1.4, Biolegend), anti-XCR1 (ZET, Biolegend), anti-CD172a (P84, BD Biosciences), anti-CD86 (GL-1, Biolegend), anti-DC-SIGN (AZN-D1, in-house), anti-CD64 (X54-5/7.1, Biolegend), anti-f4/80 (BM8, eBioscience), anti-CD45 (30-F11, Biolegend), anti-MHCII (M5/114.15, Thermofisher), anti-CD11b (M1/70, Biolegend).
