# Supplementary material for: Vaccination with DC-SIGN-Targeting αGC Liposomes Leads to Tumor Control, Irrespective of Suboptimally Activated T-Cells
Source: Pharmaceutics. 2024 Apr 24;16(5):581. doi: 10.3390/pharmaceutics16050581 (PMC11124829; doi:10.3390/pharmaceutics16050581)
Supplement: Supplementary file 1 [file pharmaceutics-16-00581-s001.zip › Supplementary Table S1.pdf]

Supplementary Table S1. Mean size, polydispersity index, and Z potential with SD of five different batches of liposomes.

Liposome content	Mean size (nm)	Mean PDI	Mean Zeta Potential (mV)
**empty**	169,1 ± 9,2	0,07 ± 0,02	-52,4 ± 6,4
**SLP**	166,3 ± 7,6	0,09 ± 0,02	-52,6 ± 8,9
**SLP-Le^{Y}**	160,9 ± 9,9	0,09 ± 0,03	-46,1 ± 5,6
**SLP-αGC**	165,2 ± 3,4	0,07 ± 0,01	-52,1 ± 4,7
**SLP-αGC-LeY**	157,1 ± 6,4	0,10 ± 0,03	-41,6 ± 5,9
